# Migalastat Treatment in a Kidney-Transplanted Patient with Fabry Disease and N215S Mutation: The First Case Report

**DOI:** 10.3390/ph14121304

**Published:** 2021-12-14

**Authors:** Valeria Di Stefano, Marta Mancarella, Antonia Camporeale, Anna Regalia, Marta Ferraresi, Marco Pisaniello, Elena Cassinerio, Federico Pieruzzi, Irene Motta

**Affiliations:** 1 UOC General Medicine, Fondazione IRCCS Ca’ Granda Ospedale Maggiore Policlinico, 20122 Milan, Italy; valeria.distefano@unimi.it (V.D.S.); marta.ferraresi@unimi.it (M.F.); elena.cassinerio@policlinico.mi.it (E.C.); 2 Department of Clinical Sciences and Community Health, University of Milan, 20122 Milan, Italy; 3 Department of Emergency Medicine, Fondazione IRCCS Ca’ Granda Ospedale Maggiore Policlinico, 20122 Milan, Italy; marta.mancarella@policlinico.mi.it; 4 Multimodality Cardiac Imaging Section, IRCCS Policlinico San Donato, 20097 San Donato Milanese, Italy; Antonia.Camporeale@grupposandonato.it; 5 Division of Nephrology, Dialysis, and Renal Transplantation, Fondazione IRCCS Ca’ Granda Ospedale Maggiore Policlinico, 20122 Milan, Italy; anna.regalia@policlinico.mi.it; 6 UOC Cardiologia, Department of Internal Medicine, Fondazione IRCCS Ca’ Granda Ospedale Maggiore Policlinico, 20122 Milan, Italy; marco.pisaniello@policlinico.mi.it; 7 Nephrology and Dialysis Unit, ASST-Monza, San Gerardo Hospital, 20900 Monza, Italy; federico.pieruzzi@unimib.it; 8 School of Medicine and Surgery, University of Milano-Bicocca, 20900 Monza, Italy

**Keywords:** Fabry disease, N215S, GLA, cardiac variant, late-onset phenotype, kidney transplant, migalastat, hypertrophic cardiomyopathy

## Abstract

Fabry disease is a rare X-linked lysosomal storage disorder caused by mutations in the GLA gene, leading to deficient α-galactosidase A activity and, consequently, to glycosphingolipid accumulation in a wide variety of cells. Fabry disease due to N215S (c.644A>G, p.Asn215Ser) missense mutation usually results in a late-onset phenotype presenting with isolated cardiac involvement. We herein present the case of a patient with N215S mutation with cardiac involvement, namely left ventricular hypertrophy and ventricular arrhythmias, and end-stage renal disease requiring kidney transplantation. To the best of our knowledge, this is the first report of a kidney-transplanted Fabry patient treated with oral pharmacologic chaperone migalastat.

## 1. Introduction

Fabry disease (OMIM #301500) is a rare X-linked lysosomal storage disorder caused by the mutation in the GLA (galactosidase alpha) gene (OMIM #300644; HGNC:4296), leading to deficient α-galactosidase A activity. The deficiency results in the progressive accumulation of globotriaosylceramide (GL-3) and its deacylated derivative globotriaosylsphingosine (lysoGb3) in lysosomes of various cells and body fluids, thereby leading to multisystemic involvement.

The pathogenesis of Fabry disease is not yet completely understood, but ischemic tissue injury consequent to the accumulation of GL-3 in vascular endothelia, particularly of small vessels, is regarded as a distinguishing feature of the disease [1]. Furthermore, the lysoGb3 seems to have cytotoxic, pro-inflammatory, and profibrotic effects [2].

The clinical manifestations are extremely variable, with the renal, neurological, cardiac, cochleovestibular, and cutaneous systems being the most involved [3]. The extent of organ involvement, the age of symptoms onset, and prognosis mainly depend on the GLA mutation, patient gender, and the degree of α-galactosidase A deficiency [4]. In addition, other genetic, epigenetic, and environmental factors may influence individual clinical presentation [5].

Hundreds of mutations in the GLA gene have been identified. Most missense mutations alter the enzyme folding and trafficking to the lysosome that lead to residual α-galactosidase A activity. This results in an atypical, late-onset phenotype with clinical manifestations confined predominantly to one organ system [1,6].

Conversely, mutations affecting the enzyme active sites result in little or no α-galactosidase A activity, which is associated with the classic phenotype in men with multisystemic manifestations [6]. However, genotype–phenotype correlation is complex because most families present a private mutation, and intra-familial phenotypic variability was previously observed [1].

N215S is a missense mutation considered responsible for late-onset cardiac phenotype since it was found in patients with predominantly cardiac manifestations, namely left ventricular hypertrophy (LVH) [7].

Treatment options for Fabry disease include enzyme-replacement therapy (ERT), which has been available since 2001, involving the replacement of the missing or deficient enzyme and, more recently, oral pharmacologic chaperone migalastat. The latter, indicated for the treatment of amenable GLA gene variants, binds to, stabilizes, and rescues misfolded forms of α-galactosidase A, leading to reduced endoplasmic reticulum retention or accumulation and facilitating proper trafficking of the enzyme to lysosomes [8].

We herein describe the case of a patient with N215S mutation, presenting with both severe cardiac and kidney involvement requiring a kidney transplant and implantable cardioverter defibrillataor (ICD) implantation. To our best knowledge, this is the first report of a case of a kidney-transplanted patient treated with migalastat.

## 2. Case Description

A 55-year-old man was first evaluated in a nephrology unit because of hypertension associated with progressive kidney failure and proteinuria. At that time, the patient presented a history of hypertension since the age of 42, hypoacusia, a superficial venous thrombosis event, and incidental evidence of cerebral ischemia.

Blood and urine tests revealed a IIIb CKD stage renal insufficiency (serum creatinine 1.92 mg/dL, MDRD GFR 39 mL/min/1.73 mq) with mild proteinuria (0.821 g/24 h). Urine sediment was clear. A kidney biopsy was performed to explore renal disease etiology, showing non-specific advanced chronic lesions (global glomerulosclerosis in 70% of glomeruli and segmental glomerulosclerosis in 10% of glomeruli, moderate interstitial fibrosis/tubular atrophy, and severe arteriolosclerosis). Therapy with ACE-inhibitor was hence started.

Two years later, at the age of 57, a significant concentric LVH (basal septum region 18 mm) was detected during an echocardiographic evaluation for hypertension.

Renal follow-up showed a progressive decline in renal function reaching end-stage renal disease (ESRD) at the patient’s age of 61. Chronic peritoneal dialysis was started, and one year later, he underwent renal transplantation from a deceased donor without complications.

When the patient was 63 years old, he began complaining of dyspnea during intense exercise. Echocardiography showed worsening LVH (maximum wall thickness of 24 mm) associated with pronounced tissue hyperechogenicity and right ventricular hypertrophy, finally raising the suspicion of Fabry disease. The diagnosis was confirmed by the detection of reduced α-galactosidase A activity measured on dried blood spots (DBS) (0.5 µmol/L/h (reference values > 2.8)) and with molecular analysis revealing the N215S GLA mutation. Increased levels of lysoGb3 on DBS (6.4 ng/mL (reference values 0.0–3.5)) were detected. At diagnosis, serum creatinine was 1.23 mg/dL, and proteinuria was 0.3 g/24 h.

Thus, in March 2019, the patient was referred to our Rare Disease Center for Fabry disease management and follow-up. The Mainz Severity Score Index (MSSI) was 40/76 (General score—3; Neurological score—3; Cardiovascular score—16; and Renal score—18), and the total assessment Fabry Disease Severity Scoring System (DS3) was 46/80 (Peripheral Nervous System domain—0; Renal domain—23; Cardiac domain—18; Central Nervous System domain—1; and Patient-Reported domain 4).

**Figure 1:**
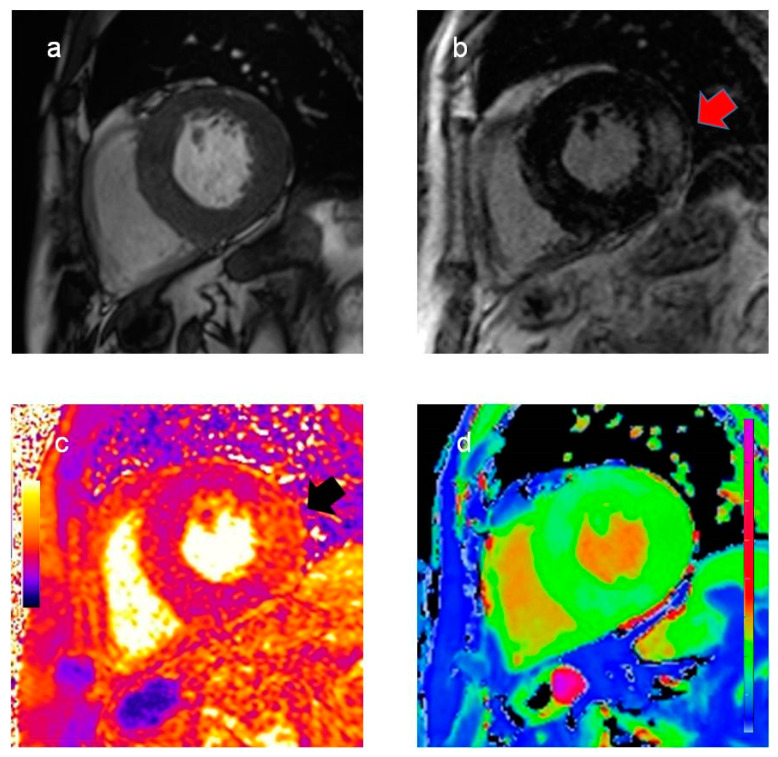
Cardiac magnetic resonance findings. Cine (**a**) and late gadolinium enhancement images (**b**), T2 (**c**), and T1 map (**d**) in basal short-axis view. Significant biventricular hypertrophy with extensive late gadolinium enhancement in the infero-lateral wall ((**b**), red arrow). Increased T2 values in the infero-lateral wall ((**c**), black arrow) indicate myocardial inflammation. Reduced native T1 values (**d**), suggesting myocardial glycosphingolipid storage, are a typical finding in Fabry disease.

Cardiac magnetic resonance (CMR) showed severe biventricular hypertrophy with reduced native T1 values, suggesting myocardial glycosphingolipid storage. The left ventricular mass index was 198.4 g/m^2^. Extensive late gadolinium enhancement was also detected in the infero-lateral wall, with an increased T2 value indicating myocardial edema (Figure 1).

An initial 24-h Holter monitoring revealed two runs of atrial fibrillation, which required the introduction of anticoagulant therapy.

One year later, in 2020, two runs of monomorphic non-sustained ventricular tachycardia were detected during a further 24-h Holter monitoring. Thus, an ICD was implanted.

Therapy with agalsidase beta at a standard dose of 1 mg/kg every two weeks, administered at our center, was initiated in June 2019. In March 2020, the outbreak of the Coronavirus pandemic dramatically affected the Northern regions of Italy. Thus, the patient asked to switch to oral therapy to avoid hospital access or contact with health care workers in the case of home treatment activation. No contraindications to chaperone therapy were found (serum creatinine 1.28 mg/dL, MDRD GFR 60 mL/min/1.73 mq). We thereby started migalastat at a standard dose of 123 mg orally every other day. The patient did not show any side effects of migalastat such as headache, runny or stuffy nose, urinary tract infection, nausea, or fever. After one year of migalastat treatment, the patient was stable in terms of symptoms, renal function (serum creatinine 1.30 mg/dL, proteinuria 0.275 g/24 h), and cardiac function (CMR in March 2021 showed substantially stable cardiac features). No adjustments in the immunosuppressor dosage, namely prednisone, mycophenolate mofetil, and tacrolimus, were required. In addition, we detected a reduction in lysoGb3 levels (4.3 ng/mL; a reduction of 33% from the patient’s basal values) and an increase in α-galactosidase A activity measured on dried blood spots (1.1 µmol/L/h; an increase of 120% compared to the patient’s basal values).

**Figure 2:**
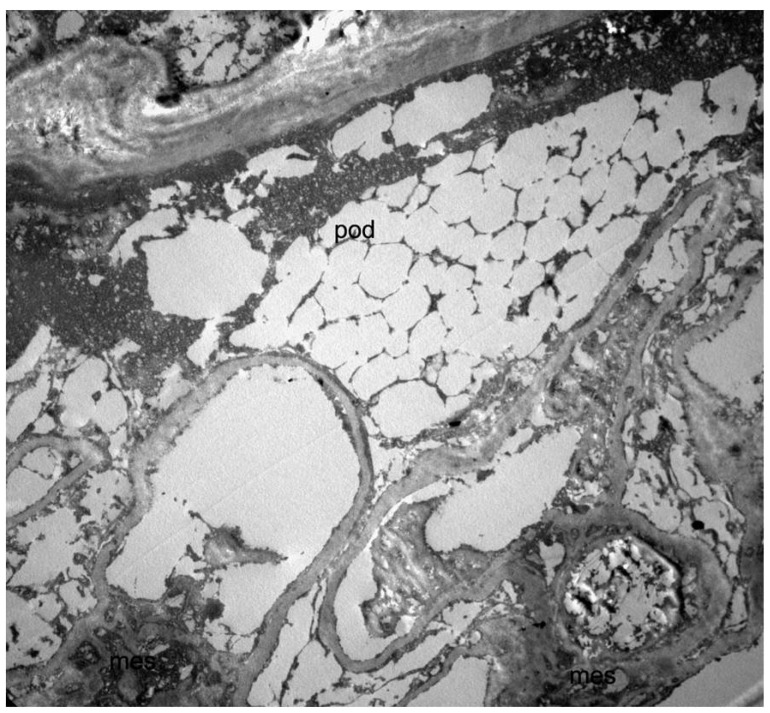
Transmission electron microscopy image of the kidney biopsy (10 × 3000), showing a multivacuolized appearance of a podocyte (pod). Since the analysis in electron microscopy was carried out on a fragment of renal tissue recovered from paraffin (and not on a standard glutaraldehyde fixed preparation), the overall structural detail was poorly preserved, and glycosphingolipid deposits could not be directly visualized due to the extraction of the lipid components. This resulted in a multivacuolized appearance of the podocytes that, although not strictly diagnostic, was compatible with the diagnosis of Fabry disease.

To confirm the kidney disease etiology, we recently analyzed the residual material from the native kidney biopsy by electron microscopy, finding abundant electron-lucent microvacuoles in many cell types and multivacuolized appearance of podocytes (Figure 2).

## 3. Discussion

N215S is a GLA missense mutation causing non-classic Fabry disease, usually presenting severe clinical manifestations that are essentially limited to the heart until late adulthood [7]. Overt renal involvement is rare, and, if present, the exclusion of non-Fabry-related medical conditions is warranted [7].

Here, we present the case of a patient with N215S mutation who presented with kidney failure at the age of 55 and ESRD at the age of 61 requiring kidney transplantation.

Germain et al. [7] analyzed the natural history in a cohort of patients with the N215S GLA mutation. At first assessment, renal involvement was present in a minority (17%) of males, with slightly abnormal estimated glomerular filtration rate (eGFR) values, most frequently among those aged 65–74 years. Moreover, severe clinical renal events (ESRD requiring renal replacement therapy) were rare (5/125), and among males, none presented a first clinical severe renal event. In a European multicenter study [9], only 1 out of 90 N215S patients presented with renal disease at a young age, with no other cause.

Finally, few cases of kidney transplant in N215S patients are described in the literature [7,10,11].

The histological hallmark of Fabry nephropathy in the N215S variant seems to be the lysosomal glycosphingolipid deposition almost exclusively within podocytes, explaining the possible presence of proteinuria without relevant kidney disfunction. Conversely, accumulation in men with the classic phenotype is detected in several renal cell types, including podocytes, mesangium, vascular endothelium, smooth muscle cells, and interstitial cells [12,13]. Interestingly, in our case, signs compatible with glycosphingolipid storage were detected in podocytes and many other renal cells, possibly explaining the atypical clinical presentation with the advanced renal disease at a relatively earlier age compared to the literature.

The presence of renal failure associated with concentric LVH should have raised suspicion of Fabry disease. As it often happens for rare diseases, it is crucial to point out the diagnostic delay. In this patient, the diagnosis was made eight years after the detection of proteinuria and kidney biopsy. Thus, we can assume that the diagnostic delay is even longer, consistently with the literature [14]. Electron microscopy (EM) was not performed at the time of biopsy, and the findings at light microscopy were interpreted as nephroangiosclerosis in a patient with hypertension. Although limited by the specimen storage conditions, findings from a retrospective overhauling of the residual material from the native kidney biopsy by EM are compatible with the kidney involvement of Fabry disease. Of note, when performing a kidney biopsy in the presence of signs and symptoms consistent with Fabry disease, EM is recommended together with light microscopy.

As expected, compared to the classical phenotype, this patient presented lower lysoGb3 concentrations [15], residual α-galactosidase A activity, and an absence of the classical Fabry disease manifestations (angiokeratoma, acroparesthesias, and cornea verticillata) [9,16,17]. Other symptoms exhibited by the patient, including hypoacusia and cerebral ischemia, can be retrospectively related to Fabry disease.

Regarding the treatment approach, he started ERT in June 2019. With the onset of the Coronavirus pandemic, intravenous ERT was shifted to oral therapy with migalastat to avoid hospitalization and contact with health care workers in an immunocompromised patient [18]. To the best of our knowledge, this is the first report of a patient who underwent a kidney transplant treated with migalastat.

Migalastat was approved in Italy in march 2017 as a first-line chaperone oral therapy in Fabry patients with amenable GLA gene variants, including the p.N215 mutation [8,19,20,21,22,23,24,25].

There is limited evidence of Fabry-specific therapy in kidney transplant recipients. Few studies showed that ERT slows the increase in, or even reduces, left ventricular mass [26,27], while no data on the effects of migalastat in this population have been reported.

According to drug product information, migalastat should not be used in patients with an eGFR < 30 mL/min per 1.73 m^2^, but there are no specific contraindications for transplanted subjects [28].

After one year of treatment, we observed stability of symptoms, renal and cardiac function, and good tolerance to therapy. Of note, no adjustments of immunosuppressive treatment were necessary.

We can conclude that migalastat administration was safe in this kidney-transplanted patient. Although data about efficacy cannot be conclusive because of the short follow-up, we noticed an increased α-galactosidase A activity related to chaperone therapy. We cannot establish the role of migalastat in reducing lyso-Gb3 levels because no analysis was performed at the treatment switch due to the Coronavirus pandemic.

In conclusion, careful monitoring of the patient is mandatory, and more data are needed to confirm the safety and evaluate the efficacy of oral chaperone therapy in kidney-transplanted patients.

## Data Availability

Data is contained within the article.
